# Local Injection of Deferoxamine Improves Neovascularization in Ischemic Diabetic Random Flap by Increasing HIF-1α and VEGF Expression

**DOI:** 10.1371/journal.pone.0100818

**Published:** 2014-06-25

**Authors:** Chen Wang, Yuanyuan Cai, Yun Zhang, Zhuyou Xiong, Guangzao Li, Lei Cui

**Affiliations:** 1 Department of Plastic and Reconstructive Surgery, Shanghai 9^th^ People's Hospital, Shanghai Jiao Tong University School of Medicine, Shanghai, P. R. China; 2 Department of Plastic and Aesthetic Surgery, Changzhou NO.2 People's Hospital, Changzhou, P. R. China; 3 Department of Plastic Surgery, 1^st^ Affiliated Hospital of Bengbu Medical College, Bengbu, Anhui, P. R. China; Indiana University College of Medicine, United States of America

## Abstract

**Background:**

Although the systemic administration of deferoxamine (DFO) is protective in experimental models of normal ischemic flap and diabetic wound, its effect on diabetic flap ischemia using a local injection remains unknown.

**Objective:**

To explore the feasibility of local injection of DFO to improve the survival of ischemic random skin flaps in streptozotocin (STZ)-induced diabetic mice.

**Methods:**

Ischemic random skin flaps were made in 125 mice. Animals were divided into the DFO-treated (n = 20), PBS-treated (n = 16) and untreated (n = 16) groups. Surviving area, vessel density, and expression of vascular endothelial growth factor (VEGF) and hypoxia-inducible factor-1α (HIF-1α) were evaluated on the seventh day after local injection.

**Results:**

The viability of DFO-treated flap was significantly enhanced, with increased regional blood perfusion and capillary density compared with those in the two control groups. Fluorescence-activated cell sorting (FACS) analysis demonstrated a marked increase in systemic Flk-1^+^/CD11b^−^ endothelial progenitor cells (EPCs) in DFO-treated mice. Furthermore, the expression of VEGF and HIF-1α was increased not only in diabetic flap tissue, but also in dermal fibroblasts cultured under hyperglycemic and hypoxic conditions.

**Conclusions:**

Local injection of DFO could exert preventive effects against skin flap necrosis in STZ-induced diabetic mice by elevating the expression of HIF-1α and VEGF, increased EPC mobilization, which all contributed to promote ischemic diabetic flap survival.

## Introduction

In diabetic patients, the management of impaired healing of cutaneous wounds, such as foot and leg ulcers, represents a significant public health burden worldwide [1], [2]. Random skin flaps are used for treating these wounds and ulcers. However, necrosis often occurs in the distal part of random skin flaps in diabetic individuals [3]–[5], which has been attributed to impaired ischemia-driven neovascularization under hyperglycemic conditions. Local administration of angiogenic growth factors, such as vascular endothelial growth factor (VEGF) [6], fibroblast growth factor-2 (FGF-2) [7], and adult mesenchymal stem cells from either bone marrow or adipose tissue, has been documented to be effective in improving neovascularization in diabetic random flaps [8], [9]. However, the safety and use of these approaches remain controversial [10], and no efficient therapy is currently available in a clinical setting.

Deferoxamine (DFO), a free radical scavenger and iron chelator, has been shown to improve skin flap survival by up-regulating VEGF in ischemic flap surgery [11]–[14]. As a key transcription factor, hypoxia-inducible factor-1α (HIF-1α) is necessary for the expression of angiogenic growth factors like VEGF, and for endothelial progenitor cells (EPCs) recruitment to ischemic sites in order to form new blood vessels [15]. Recently, it was found that the systemic use of DFO increased HIF-1α stabilization and improved age-related decline in HIF-1α [3], [11], [15]. In addition, HIF-1α activity was corrected with DFO administration by preventing iron-catalyzed reactive oxygen species (ROS) and methylglyoxal formation under hyperglycemia [3], [16], [17]. However, it is still unclear if the local administration of DFO could correct HIF-1α and VEGF expression in a hyperglycemic environment, and enhance the viability of diabetic random skin flaps.

In the present study, ischemic random skin flaps were made in streptozotocin (STZ)-induced diabetic mice, and DFO was injected locally in the skin flap to investigate whether tissue necrosis could be rescued after improved angiogenesis. Furthermore, expression of HIF-1α and VEGF was detected after DFO treatment in skin flap tissue and in skin fibroblasts cultured under high glucose and hypoxic conditions. Finally, EPCs mobilization in the peripheral circulation was evaluated in response to DFO administration.

## Materials and Methods

### Development of ischemic random skin flap in diabetic murine model

One hundred-twenty-five C57/bl6 male mice aged 8–10 weeks and weighing 20–25 g were purchased from SLAC National Rodent Laboratory Animal Resources (Shanghai, China). This study was carried out in strict accordance with the recommendations in the Guide for the Care and Use of Laboratory Animals of the National Institutes of Health. The institutional review committee of the Shanghai Jiao Tong University School of Medicine approved all animal study protocols. All mice were anesthetized with intraperitoneal injections of pentobarbital sodium (20 mg/kg body weight) in all surgical procedures, and all efforts were made to minimize suffering. Diabetes was induced by intraperitoneal injections of streptozocin (50 mg/kg, Sigma-Aldrich, St Louis, MO, USA) in 50 mM sodium citrate buffer (pH 4.5) for 5 consecutive days [18]. Venous blood was collected from the tail for blood glucose levels determination 14 days after the first day with STZ injection using a OneTouch Ultra portable glucose analyzer (Lifescan Inc., Milpitas, CA, USA). Diabetic mouse model was considered to be successful when glucose levels were above 300mg/dl and in the presence of weight loss and polydipsia, polyphagia, and polyuria symptoms.

**Figure 1:**
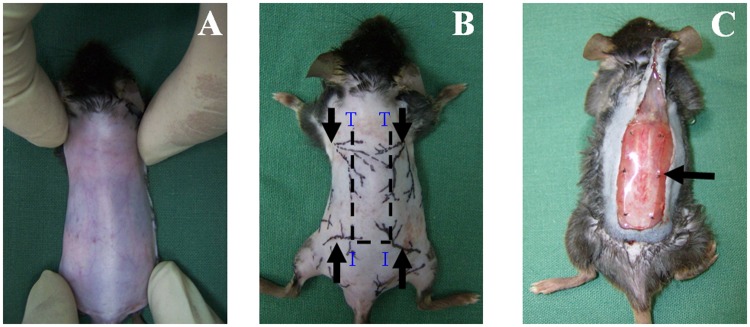
Vascular pattern of mouse dorsal skin and elevation of the skin flap. (A) The skin was gently stretched to show the cutaneous vessels. (B) Outlined cutaneous vascular architecture showing that the skin was supplied by 4 major pedicles arising from the deep circumflex iliac arteries (I) and the lateral thoracic arteries (T). (C) Ischemic flap measuring 1.0×3.0 cm was elevated and a thin silicon sheet was inserted to separate the flap from the bed. The black arrow indicates the silicone sheet.

After diabetes induction, the back's hairs of mice was cut off and soaked the remaining hair with water and daub with depilatory cream (Shibi, Shanghai, China) uniformly. Three minutes later, we erased the cream and repeated the procedure three times, the hair was removed completely and a cranially-based peninsular skin flap (1.0×3.0 cm) was elevated in the dorsum of diabetic mice [19], [20], in which the ischemic gradient was proportional to the distance from the base. The skin was gently stretched to show the cutaneous vessels (Fig. 1A), and the cutaneous vascular architecture was outlined using a marker pen (Fig. 1B). A thin sterile silicon sheet was inserted to separate the flap from the underlying bed, and the flap was sutured in place (Fig. 1C). Previous studies showed that a gradient of oxygen tensions could be generated within the flap tissue [15].

### Local injection of DFO

**Figure 2:**
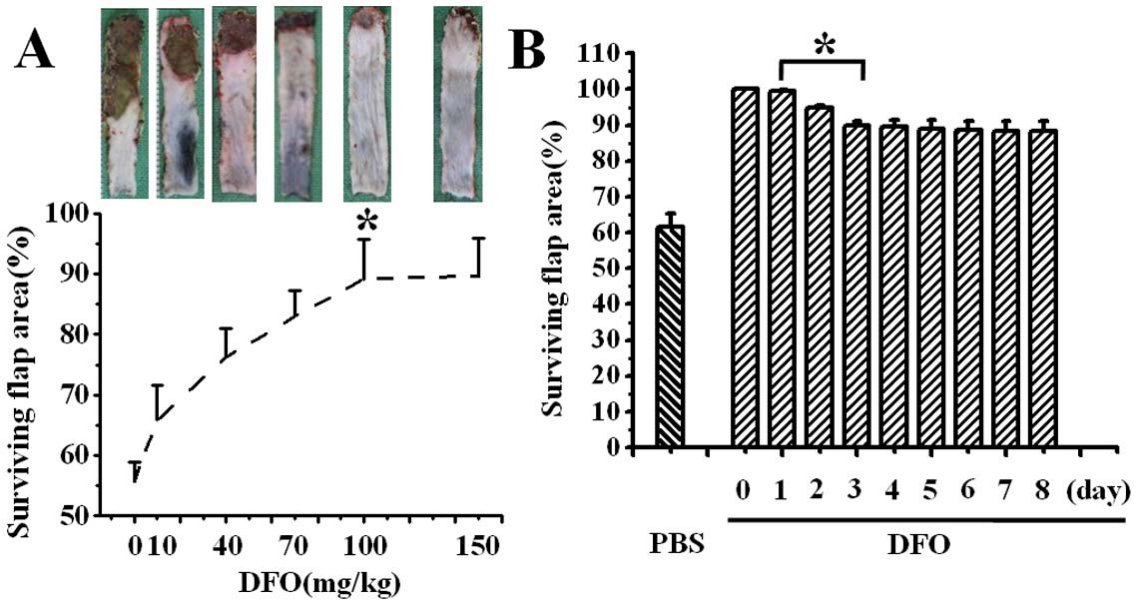
Dose- and time-dependent effects of DFO administration on the survival of diabetic skin flap. (A) Dose-dependent augmentation of surviving area of diabetic flaps upon local injection of DFO. Results are shown as means ± SEM. *P<0.01 vs. the 0–70 mg/kg groups. (B) Surviving area at different time points of diabetic flaps with injection of 100 mg/kg DFO each day. Results are shown as means ± SEM. *P>0.05. n = 6.

**Figure 3:**
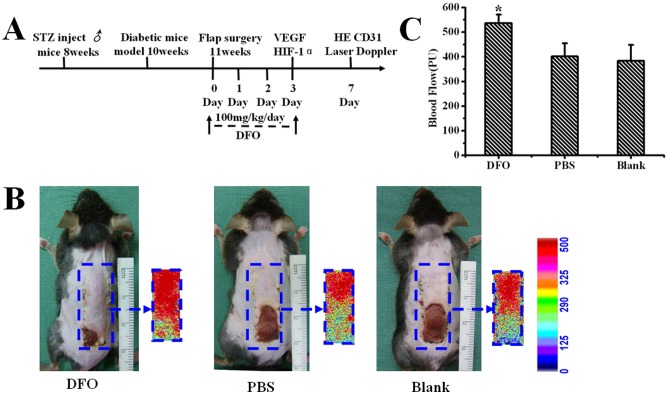
Analysis of necrosis and blood flow perfusion of the skin flap. (A) Schematic illustration of DFO injection procedure and evaluation of skin flaps. (B) Gross view and color laser Doppler detection of diabetic skin flaps from the DFO, PBS and control groups 7 days postoperation. The color scale illustrates variations in the blood flow, from maximal (red) to minimal perfusion (dark blue). (C) Quantitative analysis of blood flow perfusion of the flap measured as mean perfusion units ± SEM (*P<0.01). DFO-treated (n = 20), PBS-treated (n = 16) and Blank group (n = 16).

DFO dissolved in PBS (0.1 ml) was injected subcutaneously in the distal portion of the flap. Different concentrations of DFO (0, 10, 40, 70, 100, 150 mg/kg) were injected in the flap (Fig. 2A). The animals were divided into 3 groups: the DFO group, the PBS group and the untreated group. After flap elevation, DFO (100 mg/kg, 10 mg/ml, Sigma, St Louis, MO, USA) was immediately injected in the DFO group, and every day for three days after surgery (Fig. 3A). The same amount of PBS was subcutaneously injected in the PBS group.

### Assessment of the surviving areas of flaps and skin blood perfusion

Seven days after operation, pictures of the flaps were taken using a digital camera (HP M425, Hewlett-Packard, Palo Alto, CA, USA). Surviving area of flaps was assessed blindly by two specialists with respect to gross appearance, color, consistency of the flaps and presence or absence of bleeding. The surface area of these defined zones was then measured using the Image-Pro Plus Software 6.0 (Media Cybernetics, Silver Spring, MD, USA).

Blood perfusion of the skin flap was detected with a laser Doppler perfusion imaging system (Moor Instruments, Axminster, UK) 7 days after surgery. The probe was placed on the proximal necrosis line and 0.5 cm from the distal necrosis edge of the flap for at least 30 s, and the results were recorded as blood perfusion units (PU).

### Histology and immunofluorescence

Seven days after surgery, seven-micron-thick tissue samples were harvested from the similar position of the flaps the flap, fixed in 4% paraformaldehyde, embedded in paraffin, and stained with hematoxylin and eosin (H&E). For immunohistochemistry, samples from flap tissues were snap-frozen in liquid nitrogen; seven-micron-thick frozen sections were fixed in cold acetone and stained with rat monoclonal anti-CD31 (1∶50, ab7388, Abcam, Cambridge, MA, USA) primary antibody, followed by addition of FITC-conjugated secondary antibody (1∶1000, A-11006, Invitrogen Inc., Carlsbad, CA, USA). On one hand, neovascularization was assessed by measuring the number of capillaries in 20 fields in each mouse on H&E stained slides with high power field (HP) in each group, on the another hand, vascular density was examined under fluorescence microscope (Olympus, Tokyo, Japan) and was assessed by measuring the number of CD31+ cells in frozen sections. Capillary density was assessed morphometrically by examining three fields per section of the flap in six successive sections on both the H&E sections and the immunofluorescence staining sections after immunofluorescence staining for endothelial cells with an anti-CD31 antibody. All measurements were performed by two blinded reviewers.

### Western Blot Analysis

Cells from flaps were processed by extracting proteins with a lysis buffer (50 mM Tris-HCl, pH 7.4, containing 150 mM NaCl, 1% Nonidet P-40, 0.1% SDS, and 0.1% deoxycholate). Proteins were separated by 8% polyacrylamide gel electrophoresis containing 0.1% SDS and subsequently transferred to nitrocellulose membranes. Membranes were blocked with 2% non-fat dry milk and 3% BSA in Tris-buffered saline. Rabbit anti-HIF-1 alpha (NB100–134; Novus Biologicals, Littleton, CO, USA), or rabbit anti-VEGF (ab46154; Abcam, Cambridge, MA, USA) were added. Blots were developed using an IRDye 700DX- and IRDye 800CW-conjugated secondary antibody (Rockland Immunochemical, Inc., Gilbertsville, PA, USA), and proteins were visualized using the Odyssey system (LI-COR Biosciences, Lincoln, NE, USA).

### Culture of dermal fibroblasts

Fibroblasts were harvested from STZ-induced diabetic (n = 6, 8–10 weeks old) and non-diabetic mice (n = 6, 8–10 weeks old). Animals were sacrificed and the trunk skin was removed by sharp dissection. Special care was taken to remove the underlying adipose tissue.

Skin biopsies were harvested from diabetic and non-diabetic mice (n = 6 in each group). After being washed intensively with 0.1 M phosphate buffered saline (PBS, pH 7.4), skin tissue was minced and digested with 0.1% collagenase type I (Worthington Biochemical Corp., Lakewood, NJ, USA) at 37°C for 2 hours. Cells were then centrifuged at 600 g for 10 min and filtered through a 100-µm nylon mesh to remove undigested tissue. Cells were resuspended in Dulbecco's modified Eagle's medium (DMEM, GIBCO, Invitrogen Inc., Carlsbad, CA, USA) supplemented with 10% fetal bovine serum (FBS, Hyclone, Thermo Fisher Scientific, Waltham, MA, USA) and 1% antibiotics/antimycotics. Experiments were performed using cultured cells at the third passage. Fibroblasts from diabetic mice were maintained in high glucose medium (25 mM D-glucose) and subjected to hypoxia (1% O_2_, 5% CO_2_) to mimic the pathological diabetic parameters *in vivo* [9]. Dermal fibroblasts from diabetic or non-diabetic mice were treated with 100 µM DFO for 48 h, and cell lysates were used for western blotting [21], [22].

### Mouse EPC mobilization assay

Peripheral blood (0.4–0.6 ml) was obtained from mice in three different group (including normal phase (C57/bl6 male mice without any treatment), DM phase (diabetes was induced by intraperitoneal injections of streptozocin for 5 consecutive days) and DFO+DM phase (after diabetes induction, a skin flap was elevated in the dorsum of diabetic mice, and DFO (100 mg/kg) was injected subcutaneously in the distal portion of the flap for three days) (n = 5). Erythrocytes were lysed with ammonium chloride and separated by centrifugation. Cells were then washed with PBS/EDTA and marked using PE-labeled Flk-1 (VEGFR-2; eBioscience, San Diego, CA, USA) and FITC-labeled CD11b (eBioscience, San Diego, CA, USA) antibodies. EPCs were identified as Flk-1+/CD11b− [23]. Then, EPC mobilization was analyzed by flow cytometry (Becton Dickson, San Jose, CA, USA).

### Statistical Analysis

Results are expressed as means ± SEM. SPSS 10.0 (SPSS Inc., Chicago, IL, USA) was used for statistical analysis. Analysis of Variance (ANOVA) assuming equal variance (Student-Newman-Keul test) was performed to identify treatments different from control group, and p-values <0.05 were considered statistically significant.

## Results

### DFO improved flap viability in a dose-dependent manner

The flaps were treated with different doses of DFO and their surviving area was calculated 7 days after injection. In diabetic untreated and PBS-treated mice, severe and extended necrosis in the distal part of the flap was observed at 7 days postoperatively, accounting for 62.19±9.58% and 61.64±7.63% of the total flap area, respectively. In DFO-treated diabetic mice, there was a dose-dependent augmentation of the surviving area, being 65.83%, 83.02%, 90.10% and 89.74% at doses of 10 mg/kg, 70 mg/kg, 100 mg/kg and 150 mg/kg, respectively (P<0.01 compared with PBS-treated flaps; n = 6; Fig. 2A). In addition, there was no significant difference in surviving area between the 100 mg/kg and 150 mg/kg groups (P>0.05). Therefore, the DFO dose was fixed at 100 mg/kg for the subsequent experiments.

To find a minimal duration for injection, surviving area of skin flaps was evaluated with daily administration of DFO at 100 mg/kg for 8 days after flap elevation. The surviving area reached its peak after 2 days of DFO injection, and the subsequent DFO injections from days 3 to 8 showed no significant improvement in surviving area (Fig. 2B). This result indicated that DFO treatment initiated 1 day before and continued for 3 days after flap elevation at a dose of 100 mg/kg/day was sufficient to obtain an optimal protection against necrosis in diabetic random skin flaps.

### DFO improved flap viability by increasing blood perfusion

Using color laser Doppler imaging system analysis, it was found that blood perfusion to the distal part of the skin flaps remained lower in the untreated (396.42±14.28 PU) and PBS-treated groups (417.53±14.89 PU) 7 days after surgery, compared with DFO-treated mice (565.03±13.15 PU) (P<0.01) (Fig. 3).

### DFO improved neovascularization in ischemic skin flaps

**Figure 4:**
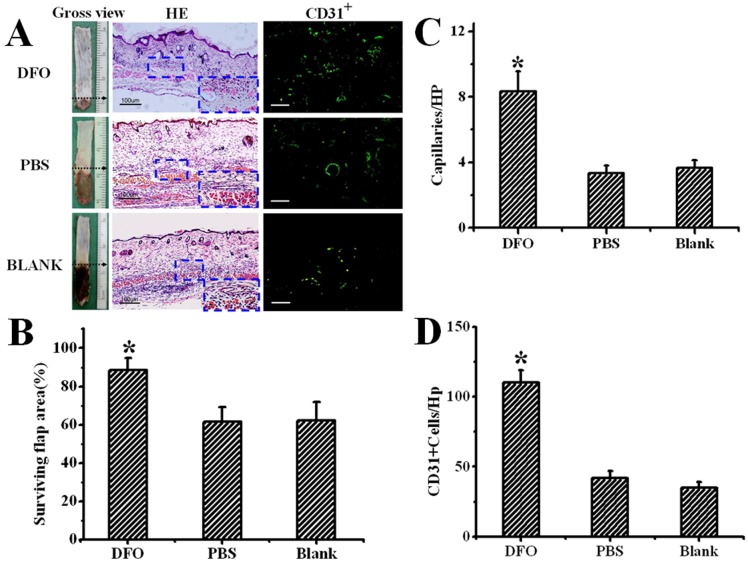
Determination of capillary density in diabetic flap 7 days after surgery. (A) Gross view, H&E staining and CD31 immunostaining of distal portions of diabetic flaps 7 days after surgery. Quantitative analysis of (B) surviving flap area, (C) capillary density, and (D) number of CD31-positive cells. Results are shown as means ± SEM. *P<0.01 vs. the PBS-treated and control groups (C and D), while *P<0.05 vs. the PBS-treated and control groups (B). Scale bar  = 100 µm. HP  =  high-power field. DFO-treated (n = 20), PBS-treated (n = 16) and Blank group (n = 16).

As it was reported that DFO promotes survival of ischemic skin flap through neovascularization, we investigated the distribution of capillaries in diabetic skin flaps. As shown in Figure 4, samples from the DFO-treated group displayed an increased distribution of capillary vessels, which exhibited dilated lumen compared with vessels in the untreated and PBS-treated groups. Furthermore, CD31-positive cells were more abundant in DFO-treated flaps.

### DFO enhanced production of VEGF and HIF-1α in diabetic skin flaps

**Figure 5:**
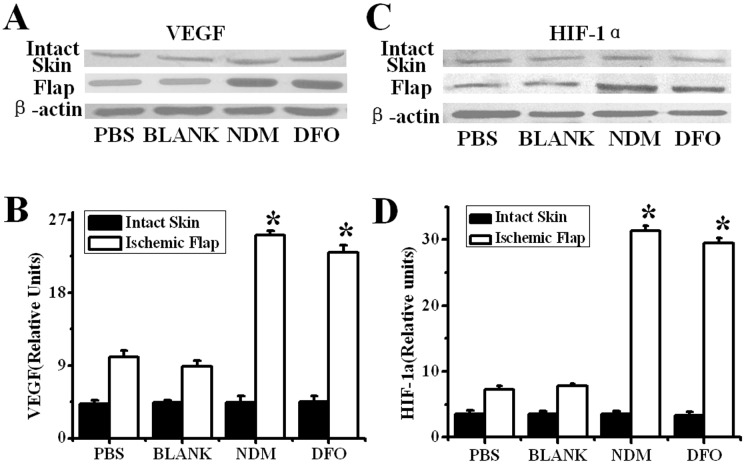
Expression of VEGF and HIF-1α in diabetic flaps evaluated by western blot 3 days after surgery. Expression of VEGF (A) and HIF-1α (C) was significantly increased in the diabetic flaps of the DFO-treated group. Semi-quantitative analysis of western blots (B and D). *P<0.01 vs. the PBS and untreated groups. NDM: Non-diabetic. n = 7.

Many studies have documented a decreased VEGF production in ischemic diabetic wounds, but it is still unclear if the improved neovascularization is the result of increased VEGF expression with administration of DFO in diabetic skin flaps. Thus, expression of VEGF and HIF-1α was examined by western blotting. As shown in Figure 5, there was no detectable difference in VEGF and HIF-1α expression in the intact skin from each group. However, compared with non-diabetic mice, the expression of VEGF and HIF-1α was dramatically decreased in untreated and PBS-treated flaps (all P<0.01). With the administration of DFO, expression of VEGF and HIF-1α was higher, and was similar to that of non-diabetic normal flaps (Fig. 5).

### DFO restored HIF-1α and VEGF expression in diabetic dermal fibroblasts

**Figure 6:**
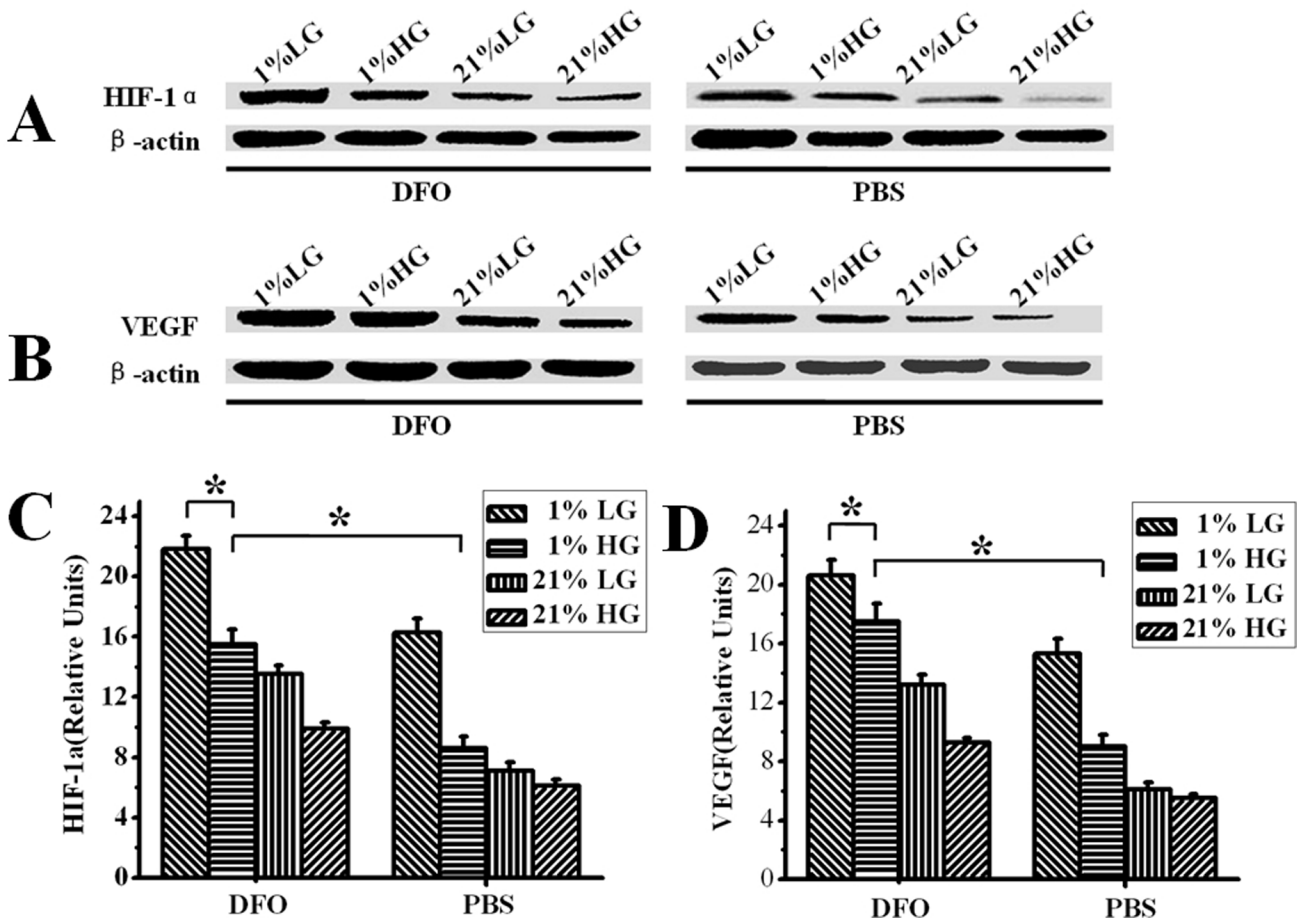
Western-blot detection of VEGF and HIF-1α production in dermal fibroblasts cultured in normoxia (21% O_2_) or hypoxia (1% O_2_) under different glucose concentrations in response to DFO administration for 72 hours. Addition of DFO in the culture medium significantly improved the expression of (A) VEGF and (B) HIF-1α in dermal fibroblasts under hypoxia and high glucose conditions. Semi-quantitative analysis of western blots (C and D). Results are shown as means ± SEM. *P<0.05. LG: Low Glucose; HG: High Glucose. n = 6.

Because dermal fibroblasts are the major actors for the healing of cutaneous wounds, the effect of DFO on HIF-1α and VEGF expression in dermal fibroblasts cultured under high glucose and hypoxic conditions was assessed. Fibroblasts were grown in either low (5 mM D-glucose) or high glucose (25 mM D-glucose) medium for 2 weeks, and were then exposed to normoxic or hypoxic conditions for 3 days. Under hypoxic conditions, DFO treatment resulted in a 1.7-fold and 1.8-fold increase in HIF-1α and VEGF expression, respectively, in cells cultured in high glucose medium, approximating the levels in cells cultured in low glucose medium (Fig. 6). Under normoxia (21% O_2_), HIF-1α and VEGF levels in cells cultured in high glucose medium showed an increase that was similar to the cells cultured in low glucose medium with the DFO administration (Fig. 6A, C).

### DFO promoted increased EPC mobilization

**Figure 7:**
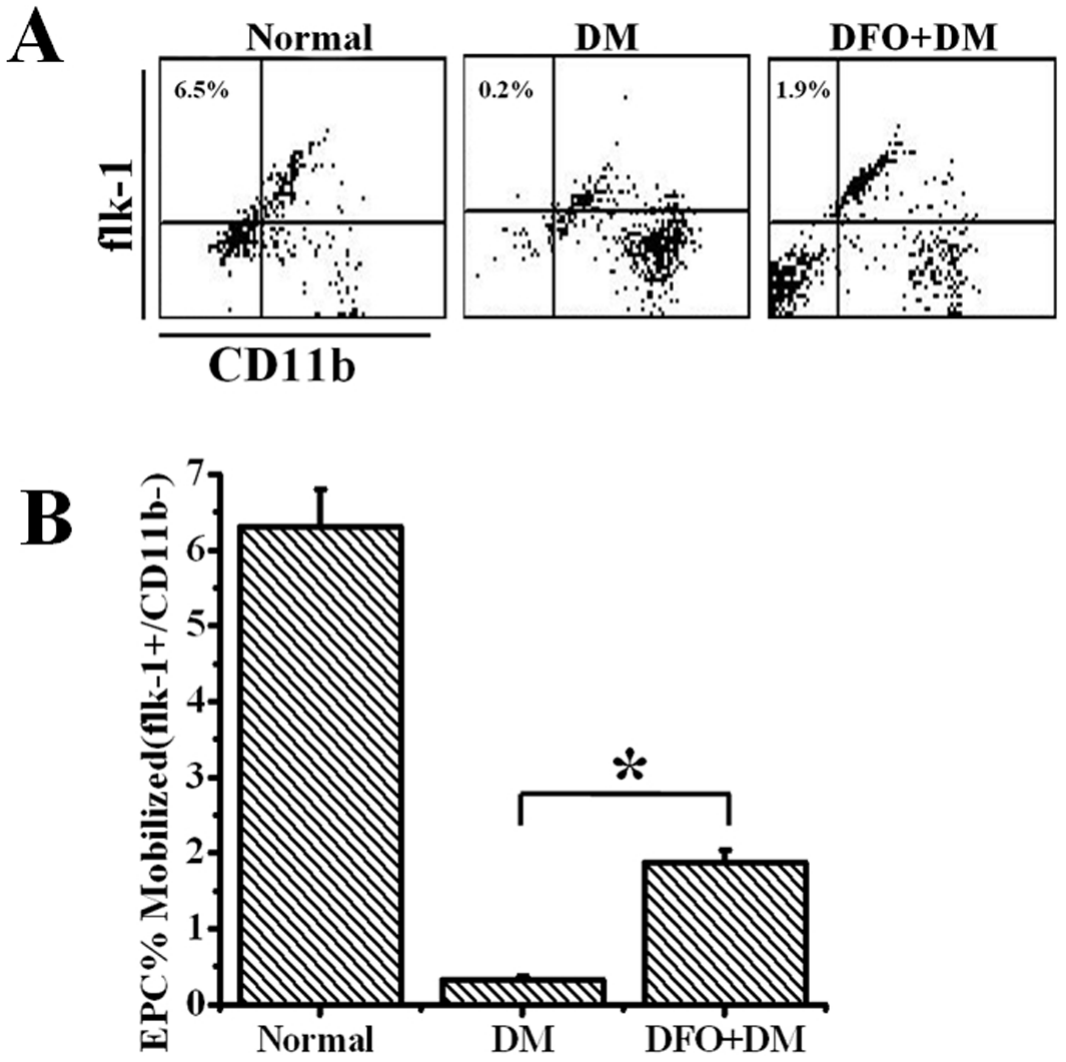
Proportion of circulating EPCs in DFO-treated and untreated diabetic mice, as determined by FACS analysis (A). Flk1^+^/CD11b^−^ cells represent circulating EPCs. (B) Quantitative analysis of EPC mobilization are shown as means ± SEM. *P<0.05. DM: diabetic. Normal indicates mice without any treatment. n = 5.

To examine whether diabetic EPCs retained the ability to respond to DFO, EPCs from the peripheral blood were analyzed by FACS. Diabetic mice exhibited a marked decrease in systemic Flk-1^+^/CD11b^−^ progenitor cells compared with non-diabetic mice, indicating a specific impairment of vasculogenesis in diabetic mice (6.34±0.32% vs. 0.42±0.06%, P<0.01; Fig. 7B). With DFO treatment, FACS analysis demonstrated a marked increase in systemic Flk-1^+^/CD11b^−^ EPCs in diabetic mice compared with controls (1.85±0.12% vs. 0.42±0.06% EPCs, P<0.05) (Fig. 7A–B). Thus, increased EPC mobilization was induced by DFO administration and may contribute in part to the increased neovascularization of the ischemic flap in diabetic mice.

## Discussion

In the present study, we showed that the local injection of DFO improved the viability of random skin flaps in STZ-induced diabetic mice, which was accompanied by increased capillary density in necrotic area. Expression of VEGF and HIF-1α was also increased by the administration of DFO. Furthermore, local injection of DFO resulted in an increased mobilization of EPCs in diabetic mice.

Necrosis of skin flaps in diabetic conditions has been attributed to chronic hyperglycemia, which leads to defective angiogenesis in tissues responding to low oxygen tension [9]. Thus, restoring adequate response to hypoxia in ischemic tissue is of vital importance to stimulate neovascularization in the flap. In the present study, we showed that local administration of DFO significantly increased the viability of random skin flaps in diabetic mice. Meanwhile, within the DFO-treated diabetic flaps, an elevated blood perfusion was detected, which was concordant with enhanced capillary density determined by CD31 immunofluorescence. Furthermore, mobilization of EPCs in the peripheral circulation was greatly increased in DFO-treated diabetic mice compared with the control groups. Taken together, these results demonstrated that improved viability of diabetic skin flaps by local administration of DFO was a consequence of stimulated neovascularization.

As the key transcription factor that mediates the adaptive response to hypoxia, HIF-1α has been proved to be a key regulator of angiogenic gene expression by binding to a conserved and defined hypoxia response element in the genes activated by it [24], [25]. However, it was found that high-glucose conditions induced the production of methyglyoxal, leading to the modification of HIF-1α coactivator p300, which attenuates the association of p300 with HIF-1α, and therefore prevents HIF-1-mediated gene transactivation. Therefore, DFO could attenuate methylglyoxal production, decrease the modification of p300 by methylglyoxal, and thus normalize hypoxic response of cells exposed to high glucose environment by the correction of impaired HIF-1α/p300 association [3]. However, whether the improved neovascularization induced by DFO in diabetic flaps is the result of increased HIF-1α accumulation remains unclear. In the present study, we found that the expression of HIF-1α was greatly reduced in diabetic flaps. With the local administration of DFO, the expression of HIF-1α and VEGF recovered to a similar level as that in non-diabetic mice, which was in accordance with improved viability of skin flaps. It has been reported that hyperglycemia attenuates VEGF production [2], [3], and decreased VEGF levels have been observed in the wounds of diabetic mice [2], [26]. Thus, with improved HIF-1α expression, VEGF production was restored by local DFO injection.

The repair and regeneration of the vascular system requires local vessel remodeling through vasculogenesis and angiogenesis [15], during which EPCs are mobilized to the ischemic sites from the bone marrow and take part in the formation of new blood vessels in injured tissue [27]. Vasculogenesis requires a subtle cascade of signaling events capable of mobilizing, homing and retaining bone marrow-derived EPCs to induce neovascularization in response to up-regulated HIF-1α and local VEGF production [28]. However, in agreement with the present study, previous studies have demonstrated that mobilization of EPCs is greatly decreased in diabetic mice [29], [30]. Therefore, the necrosis of diabetic flaps may be secondary to decreased EPC recruitment as a result of decreased HIF-1α and VEGF expression. The ability to stabilize HIF-1α with DFO administration and to retain a normal hypoxia response is critical for the guidance and retention of reparative EPCs. With DFO administration, angiogenesis in ischemic diabetic random flaps was improved as a result of stabilization of intracellular HIF-1α in response to hypoxia, by which VEGF production and EPCs migration was enhanced [31]. These results were consistent with the findings by Chang et al, who reported that DFO only promoted neovascularization in ischemic areas, and did not produce any neovascularization in uninjured skin [15].

Dermal fibroblasts play a critical role for regulating contraction, extracellular matrix deposition and neovascularization in cutaneous wound repair. Lerman et al. revealed that dermal fibroblasts from diabetic mice exhibited impaired migration, VEGF expression and response to hypoxia under hyperglycemia [32]. In the present study, we observed that diabetic fibroblasts failed to produce HIF-1α and VEGF in response to hypoxia. However, with the addition of DFO in the culture medium, expression of HIF-1α and VEGF was restored to the same levels as non-diabetic fibroblasts. Thus, this *in vitro* study suggested that DFO performed its angiogenic role through the increase of HIF-1α and VEGF expression in dermal fibroblasts in necrotic flaps in diabetic mice.

Many findings have reported that DFO could stabilize HIF-1α and increase VEGF expression [11], [15], [33]. As a result, DFO promoted neovascularization in normal skin flaps in aged mice and enhanced wound healing in diabetic tissues [3], [11], [15]. To our knowledge, this is the first report about the improvement of the survival of diabetic skin flaps by local administration of DFO. The local delivery of DFO for the treatment of human diabetic necrotic flaps may have significant clinical implications for diabetic patients [34], [35]. Of course, further studies should focus on DFO-mediated cellular cascade involving eNOS, SDF-1α, and PHD, which play roles in the neovascularization in ischemic environment and in diabetic-related complications [30], [36], [37].

## Conclusion

In conclusion, our studies demonstrated that a local injection of DFO could improve the viability of ischemic random skin flaps by enhancing neovascularization in STZ-induced diabetic mice. With DFO administration, HIF-1α stability was increased and led to an increased expression of VEGF, as well as promoted the mobilization of EPC in the peripheral circulation. In addition, DFO restored HIF-1α-mediated VEGF expression in diabetic fibroblasts in response to hypoxia. Thus, local administration of DFO may be a safe, easy and alternative treatment for necrotic random skin flap in diabetic patients.
